# Selective targeting of KRAS-driven lung tumorigenesis via unresolved ER stress

**DOI:** 10.1172/jci.insight.137876

**Published:** 2021-04-08

**Authors:** Iwao Shimomura, Naoaki Watanabe, Tomofumi Yamamoto, Minami Kumazaki, Yuji Tada, Koichiro Tatsumi, Takahiro Ochiya, Yusuke Yamamoto

**Affiliations:** 1 Division of Cellular Signaling, National Cancer Center Research Institute, Chuo-ku, Tokyo, Japan.; 2 Department of Respirology, Graduate School of Medicine, Chiba University, Chuo-ku Chiba-shi, Chiba, Japan.; 3 Department of Respiratory Medicine, International University of Health and Welfare Atami Hospital, Atami-shi, Shizuoka, Japan.; 4 Department of Molecular and Cellular Medicine, Institute of Medical Science, Tokyo Medical University, Shinjuku-ku, Tokyo, Japan.

**Keywords:** Cell Biology, Oncology, Drug screens, Lung cancer, Oncogenes

## Abstract

Lung cancer with oncogenic *KRAS* makes up a significant proportion of lung cancers and is accompanied by a poor prognosis. Recent advances in understanding the molecular pathogenesis of lung cancer with oncogenic *KRAS* have enabled the development of drugs, yet mutated *KRAS* remains undruggable. We performed small-molecule library screening and identified verteporfin, a yes-associated protein 1 (YAP1) inhibitor; verteporfin treatment markedly reduced cell viability in KRAS-mutant lung cancer cells in vitro and suppressed KRAS-driven lung tumorigenesis in vivo. Comparative functional analysis of verteporfin treatment and YAP1 knockdown with siRNA revealed that the cytotoxic effect of verteporfin was at least partially independent of YAP1 inhibition. A whole-transcriptome approach revealed the distinct expression profiles in KRAS-mutant lung cancer cells between verteporfin treatment and YAP1 knockdown and identified the selective involvement of the ER stress pathway in the effects of verteporfin treatment in KRAS-mutant lung cancer, leading to apoptotic cell death. These data provide novel insight to uncover vulnerabilities in KRAS-driven lung tumorigenesis.

## Graphical abstract

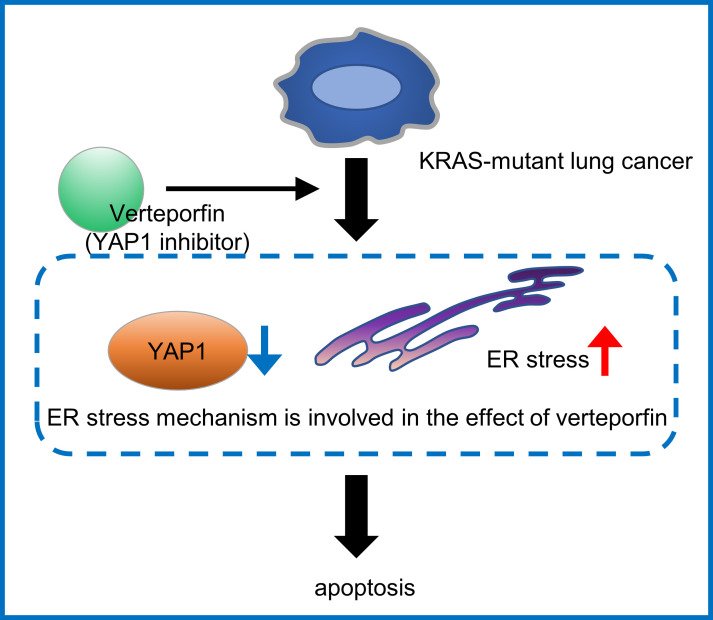

## Introduction

Lung cancer is the leading cause of cancer death worldwide, and an estimated more than 1 million deaths due to cancer occur per year (1, 2). Unfortunately, the prognosis of lung cancer remains dismal, with a 5-year survival rate of approximately 15% (3). New advances in the discovery of molecular targeted therapies against oncogenic drivers have brought robust breakthroughs, yet activating mutations of *KRAS* remain undruggable targets (4, 5). Major pathways, such as the RAF/MEK/ERK and PI3K/AKT/mTOR networks, are regulated by activated *KRAS* for the progression of cancer survival. As crucial components of the struggle against lung cancer, a better understanding of cancer biology and an increase in the population that benefits from cancer therapeutics are required.

The Hippo pathway, which was first discovered on the basis of tissue growth in *Drosophila melanogaster*, is a powerful regulator that controls organ growth, cell differentiation, and tissue homeostasis (6). The highly related transcriptional regulators yes-associated protein (YAP) and transcriptional co-activator with PDZ-binding motif (TAZ) are a fundamental source of structural and architectural features during cellular proliferation and differentiation (7, 8). YAP/TAZ have attracted much interest in recent years as triggers of several hallmarks of cancer, and YAP/TAZ activity has been shown to be essential for development, progression, and metastasis (9). Recent studies have linked the complexity of YAP/TAZ in cancer with other cancer-relevant factors and pathways, such as KRAS, APC, LKB1, aberrant GPCR signaling, and WNT signaling (10). In lung cancer, aberrant expression of YAP is correlated with resistance to therapeutic drugs, cancer progression, and metastasis to distant sites, such as the lymph node and brain (11, 12). Deregulation of the Hippo pathway, which is mostly carried out by YAP in the nucleus, was shown to induce a growth regulation pathway in the nucleus in approximately 65% of non–small cell lung cancer (13). Furthermore, elevated YAP expression in lung cancer patients has been associated with poor prognosis (9, 14). Despite these recent advances in understanding YAP in the cancer field, the function of YAP in cells or tissues in lung cancer tumorigenesis remains to be explored. Verteporfin, a light-activated compound used in photodynamic therapy for ophthalmic disorders, has recently been proved to be an antitumor modality via blocking the interaction between YAP and transcriptional enhanced associated domain (TEAD) (15). It was also reported that combinatorial therapy with pan-RAF inhibitors and YAP inhibitor had synergistic effects on KRAS-mutant pancreatic cancer (16); on the other hand, the antitumor activity of verteporfin in colon cancer was demonstrated to be independent of YAP (17). Since the reliability of verteporfin as a specific YAP inhibitor remains controversial, further exploration of the mechanism of verteporfin is warranted.

In this study, drug screening in KRAS-mutant lung cancer cells identified verteporfin as a specific therapeutic candidate. We explored the biological mechanism of the antitumor activity of verteporfin in comparison with YAP1-knockdown experiments. Using this approach, we also investigated whether YAP1 itself is the key determinant of KRAS-driven lung tumorigenesis.

## Results

### Identification of verteporfin as a cytotoxic agent in KRAS-mutant lung cancer cells.

**Figure 1:**
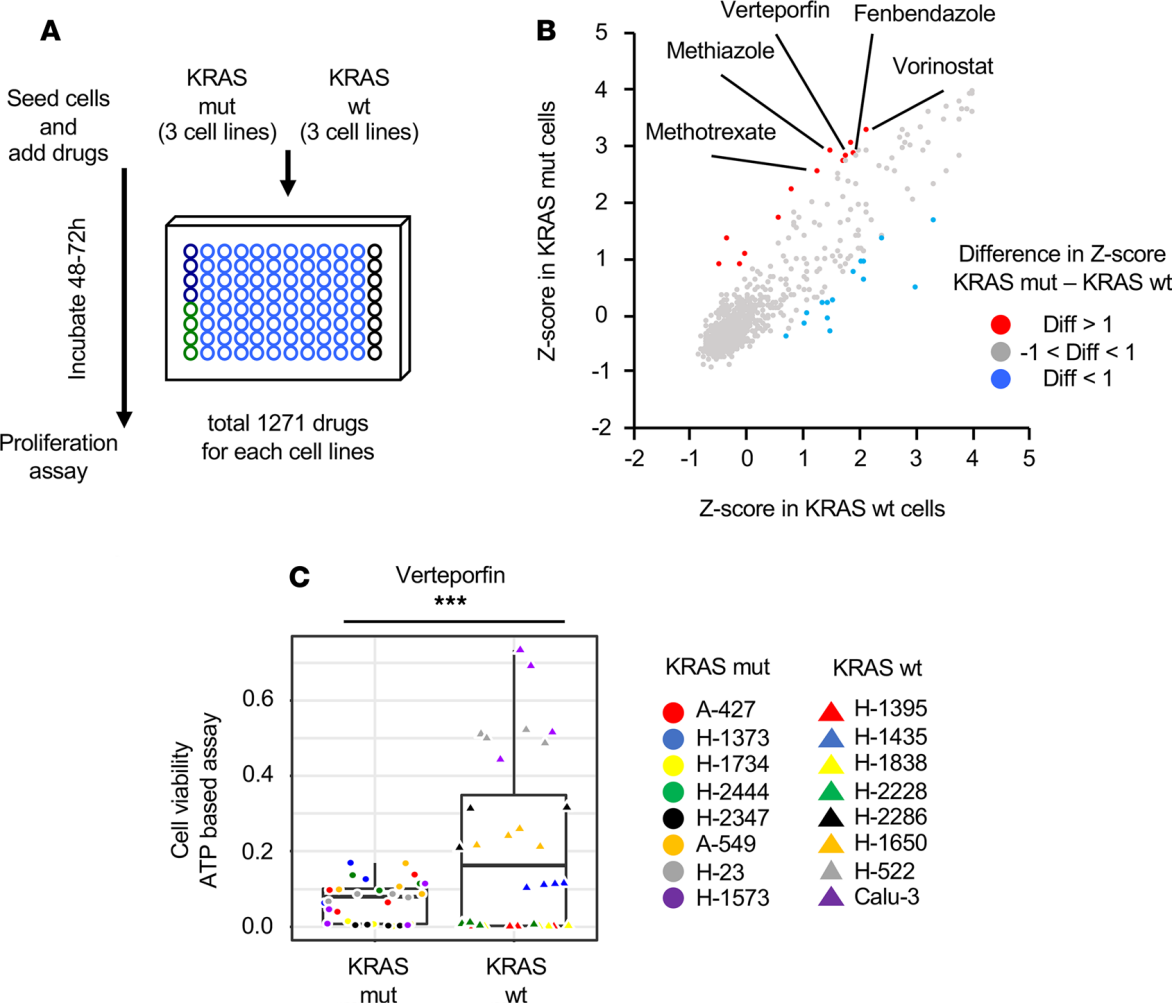
Verteporfin inhibits KRAS-mutant lung cancer cell growth. (**A**) Scheme of the protocol used to screen 1271 drugs. (**B**) Scatterplot of the difference in *z* score between KRAS-mutant cells and WT cells. (**C**) Quantification of cell viability in KRAS-mutant and WT cells after treatment with verteporfin. The values are the mean ± SD (8 KRAS-mutant cell lines and 8 WT cell lines, each *n* = 4). Statistical significance was determined using an unpaired 2-tailed Student’s *t* test. ***, *P* < 0.001.

With a previously published protocol (18), we sought to identify a specific inhibitor of KRAS-mutant lung cancer cells and assessed drug sensitivity by small-molecule screening (Figure 1A). As a screen, we used the Prestwick Chemical Library of 1271 small molecules in clinical use. Further methods and results of the screening have been shown in our previous report (18). We measured the cell viability, which is represented by *z* score, and determined the difference in cell viability between KRAS-mutant lung cancer cells and WT cells (Figure 1B). Among them, verteporfin, known to be a YAP1 inhibitor, was selected as a candidate (Table 1). Verteporfin was selected as a candidate because (a) the molecular structure of verteporfin is unique (Supplemental Figure 1A; supplemental material available online with this article; https://doi.org/10.1172/jci.insight.137876DS1), and (b) verteporfin was recently reported to be a YAP1 inhibitor with an inhibitory effect on oncogenic growth in various cancers (15, 19, 20). Verteporfin, a porphyrin derivative, was recently shown to have antitumor effects in cancer cells in the absence of light-mediated activation (21). We focused on the treatment effects of verteporfin in KRAS-mutant lung cancer cells. Further validation assays using 16 cell lines (8 KRAS-mutant cell lines and 8 WT cell lines) supported the effect of verteporfin as a specific tumor growth inhibitor of KRAS-mutant lung cancer cells (Figure 1C and Supplemental Figure 1B). We analyzed the gene expression profile of Hippo pathway–related genes in all cell lines according to a public database (22, 23). There was no significant difference in these gene expression levels, although YAP1 expression was generally high in most of the cell lines (Supplemental Figure 1C). Further analysis of caspase-3/7 activity in the 16 cell lines (8 KRAS-mutant cell lines and 8 WT cell lines) showed that verteporfin increased apoptotic cell death in KRAS-mutant cells compared with WT cells (Supplemental Figure 1D). Thus, we found that verteporfin has remarkable cytotoxic effects in KRAS-mutant cells.

### Verteporfin suppressed KRAS-driven lung tumorigenesis in vivo.

**Figure 2:**
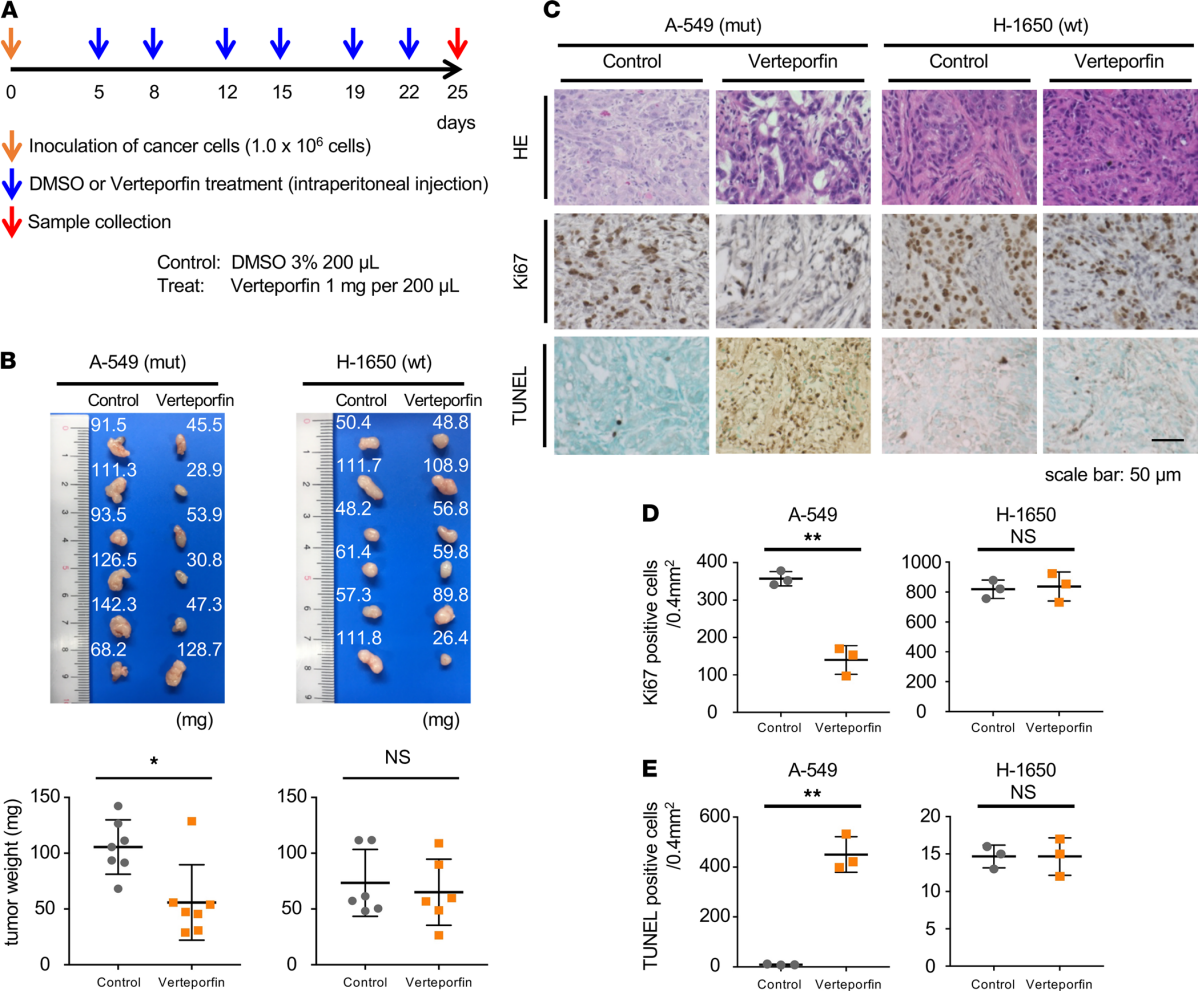
Verteporfin exerts apoptotic effects in KRAS-mutant cells in vivo. (**A**) Schematic timeline of the animal study. (**B**) Representative pictures of tumors harvested from KRAS-mutant xenograft mice and WT xenograft mice. Tumor weight was measured at the time of sample collection. The values are the mean ± SD (*n* = 6). Statistical significance was determined using an unpaired 2-tailed Student’s *t* test. *, *P* < 0.05. (**C**) Representative histology of H&E-stained sections, Ki67-stained sections, and TUNEL-stained sections of tumors from KRAS-mutant xenograft mice and WT mice. Quantification of the percentage of Ki67-positive (**D**) and percentage of TUNEL-positive (**E**) cells. The values are the mean ± SD (*n* = 3). Statistical significance was determined using an unpaired 2-tailed Student’s *t* test. **, *P* < 0.01.

To examine the effect of verteporfin on KRAS-mutant cells in vivo, we utilized a subcutaneous xenograft mouse model that harbored either KRAS-mutant cells or WT cells. When tumors became palpable, mice were randomized to a verteporfin treatment group and a vehicle-treated control group. According to the protocol shown (Figure 2A), verteporfin was intraperitoneally injected into mutant (A-549) xenograft mice twice a week for a total of 3 weeks. Overall, tumor growth was strongly inhibited by the treatment with verteporfin, while no significant difference in tumor growth was observed in KRAS WT (H-1650) xenograft mice (Figure 2B). Histopathological examination indicated that the tumors treated with verteporfin exhibited nuclear aggregation and cell fragmentation (Figure 2C, upper). Assessment of Ki67 in KRAS-mutant xenografted mice indicated significantly less cell proliferation compared with that in the control tumors (Figure 2C, middle, and Figure 2D). The expression of apoptosis markers (assessed with TUNEL staining) was remarkably different in the verteporfin treatment group than in the control group of KRAS-mutant xenograft mice (Figure 2C, bottom, and Figure 2E). Because in vitro measurements of caspase 3/7 showed that verteporfin induced a significant increase in apoptosis in normal epithelial cells (Supplemental Figure 2A), we expected that verteporfin treatment in vivo would cause severe side effects in the mice; however, no significant differences in body weight or the results of serological biochemical tests between the treatment and the control groups of both KRAS-mutant and WT xenograft mice were observed (Supplemental Figure 2, B and C). In summary, these data indicate that the verteporfin treatment inhibits tumor growth and induces apoptosis of KRAS-mutant tumors in vivo without severe side effects.

### The effects of YAP1 knockdown do not simply mirror verteporfin treatment.

**Figure 3:**
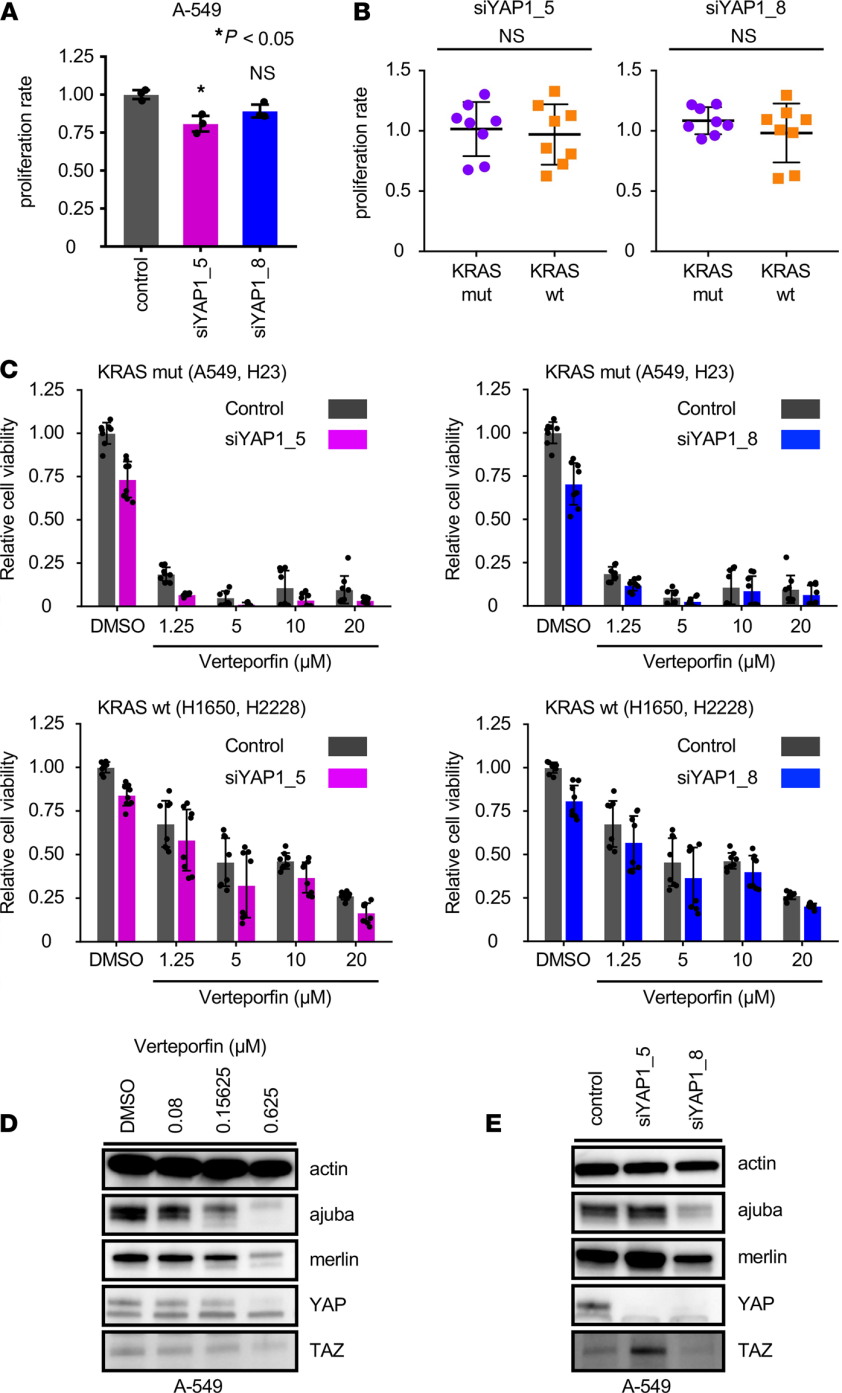
Verteporfin treatment and YAP1 knockdown have different effects on cell proliferation and YAP1 expression. (**A**) Effects of YAP1 siRNAs on the proliferation of A-549 cells. The values are the mean ± SD. Statistical significance was determined using Dunnett’s multiple-comparison test. *, *P* < 0.05. (**B**) Validation experiments using 8 KRAS-mutant cell lines and 8 WT cell lines in which YAP1 was knocked down by siRNAs. The values are the mean ± SD (8 KRAS-mutant cell lines and 8 WT cell lines, each *n* = 4). Statistical significance was determined using an unpaired 2-tailed Student’s *t* test. (**C**) Effects of combination treatment of verteporfin and YAP siRNA knockdown in KRAS-mutant and WT cells. The values are the mean ± SD (each, *n* = 4). (**D**) Western blots from A-549 cells after treatment with verteporfin. (**E**) Western blots from A-549 cells after treatment with YAP1 siRNAs.

Given that verteporfin inhibits cell proliferation in KRAS-mutant lung cancer cells in vitro and in vivo and that the effects of verteporfin through YAP1 inhibition are still controversial, we evaluated whether the inhibitory effect of verteporfin on cell proliferation was truly mediated by YAP1 inhibition. To this end, cell proliferation following YAP1 knockdown was examined in KRAS-mutant and WT lung cancer cells using 2 YAP1 siRNAs, siYAP1-5 and siYAP1-8 (Supplemental Figure 3A). Although both YAP1 siRNAs (siYAP1-5, siYAP1-8) inhibited cell proliferation, the inhibitory effects were drastically small compared with those of verteporfin treatment (Figure 3A and Supplemental Figure 3B). The difference in proliferation due to YAP1 knockdown was not significant between KRAS-mutant and WT cells transfected with both siRNAs (Figure 3B), and there was no correlation between verteporfin treatment effects (Supplemental Figure 3C). We examined the effects of verteporfin in YAP1-knockdown cells to evaluate whether verteporfin possesses a YAP1-independent mechanism. Cell proliferation was inhibited by verteporfin treatment even in YAP1-knockdown cells both in KRAS-mutant and in WT cells (Figure 3C). These results showed the presence of a YAP1-independent mechanism in verteporfin treatment. Next, the expression of proteins related to the Hippo pathway after verteporfin treatment in A-549 cells was evaluated by Western blotting, and as expected, their protein levels decreased in a dose-dependent manner (Figure 3D). Compared with verteporfin treatment, YAP1 knockdown obviously inhibited YAP1, although other components showed only a clear decease in cells after siYAP1-8 treatment (Figure 3E).

### Transcriptomic profile of verteporfin treatment and YAP knockdown.

**Figure 4:**
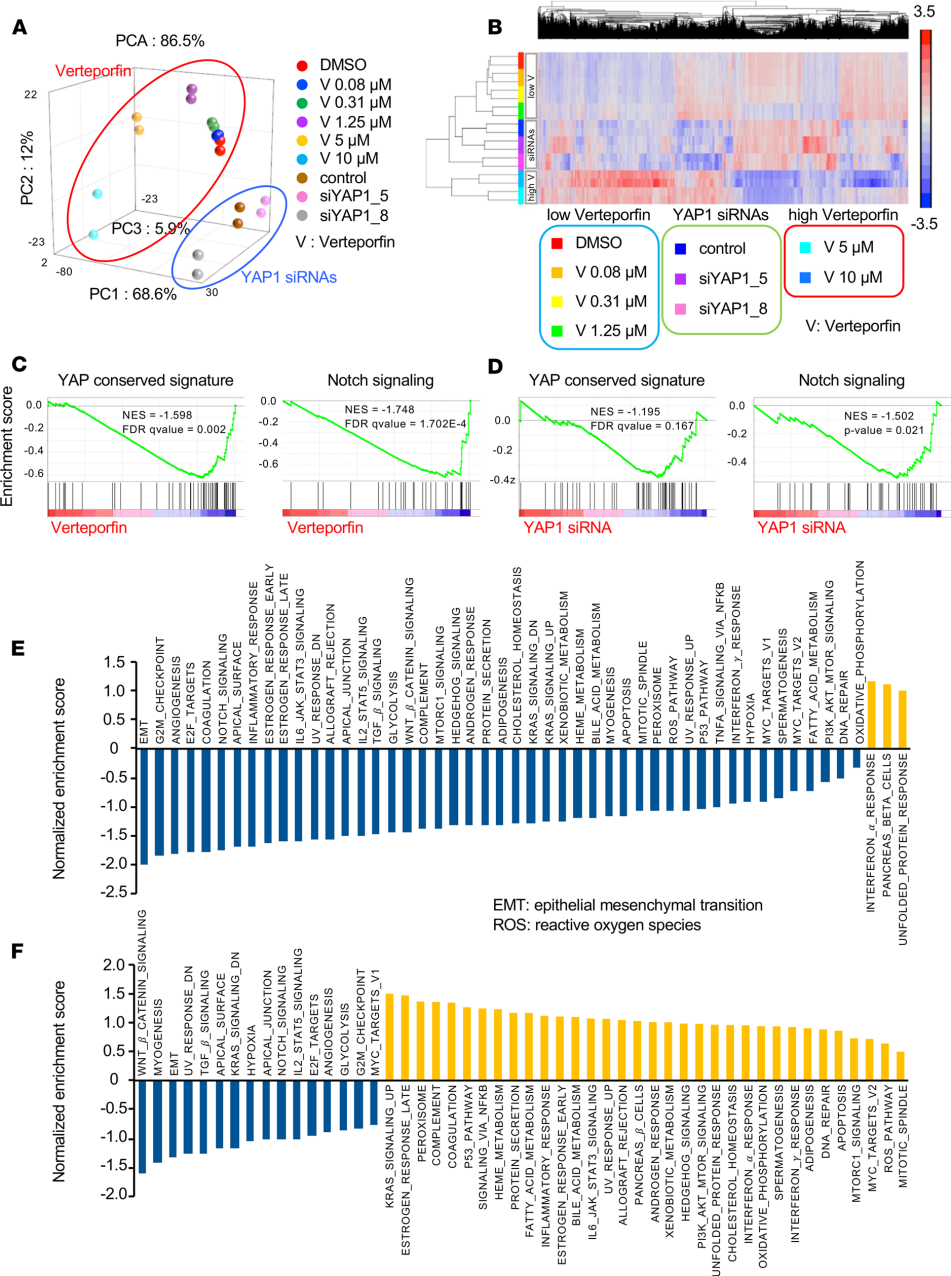
A whole-transcriptome analysis in KRAS-mutant lung cancer cells between verteporfin treatment and YAP1 knockdown. (**A**) Principal component analysis (PCA) of A-549 cells treated with YAP1 siRNA and verteporfin at various concentrations. (**B**) Heatmap showing the upregulated and downregulated genes following treatment with YAP1 siRNAs and verteporfin at various concentrations. (**C**) GSEA of the A-549 cells treated with verteporfin at various concentrations. NES, normalized enrichment score. The *P* values in the graphs were calculated by GSEA. (**D**) GSEA of the A-549 cells treated with YAP1 siRNAs. The *P* values in the graphs were calculated by GSEA. (**E**) Normalized enrichment scores from GSEA of all hallmark gene sets by following verteporfin treatment. EMT, epithelial-mesenchymal transition. (**F**) Normalized enrichment scores from GSEA of all hallmark gene sets by YAP1-knockdown experiments.

Recent studies have indicated the inconsistent effects of RNA interference and small-molecule inhibition on oncogenic KRAS-driven lung cancer cells (24). Researchers have concluded that this discrepancy is due to the lower specificity of the small molecules. To further determine the effects of verteporfin in KRAS-mutant lung cancer cells, we interrogated cells treated with verteporfin at a range of doses by RNA-Seq. Whole-transcriptome analysis of the effects of verteporfin treatment and YAP1 knockdown found that the differentially expressed genes were separately clustered as different subsets (Figure 4, A and B). According to whole-transcriptome data, the gene expression patterns also varied in a dose-dependent manner, as shown by PCA mapping (Supplemental Figure 4A). A large number of genes affected by verteporfin treatment are shown in Supplemental Figure 4B, and their expression also differed in a dose-dependent manner. Gene set enrichment analysis (GSEA) revealed a conserved signature YAP pathway was significantly suppressed by verteporfin treatment (Figure 4C). Other cancer-related pathways, such as Notch signaling, were also influenced by verteporfin, which is consistent with previous reports (Figure 4C) (25). RNA-Seq of YAP1 knockdown cells showed that treatment with both siRNAs produced different gene expression patterns in PCA maps from those of control samples (Supplemental Figure 4C). The differentially expressed genes are shown in heatmaps (Supplemental Figure 4D) and were considered with the results of treatment with the 2 siRNAs for further analysis. GSEA also showed that verteporfin treatment has similar effects on YAP1 and Notch signaling (Figure 4D). On the other hand, analysis of the pathways included in the hallmark gene sets revealed that YAP1 knockdown and verteporfin treatment had obviously different influences on these gene pathways (Figure 4, E and F). These results indicate that treatment with verteporfin and YAP1 knockdown have some similar effects on YAP1 signaling pathways but also exhibit clear differences that might contribute to the cytotoxicity of verteporfin in KRAS-mutant lung cancer cells.

### Verteporfin induces apoptosis via ER stress pathway.

**Figure 5:**
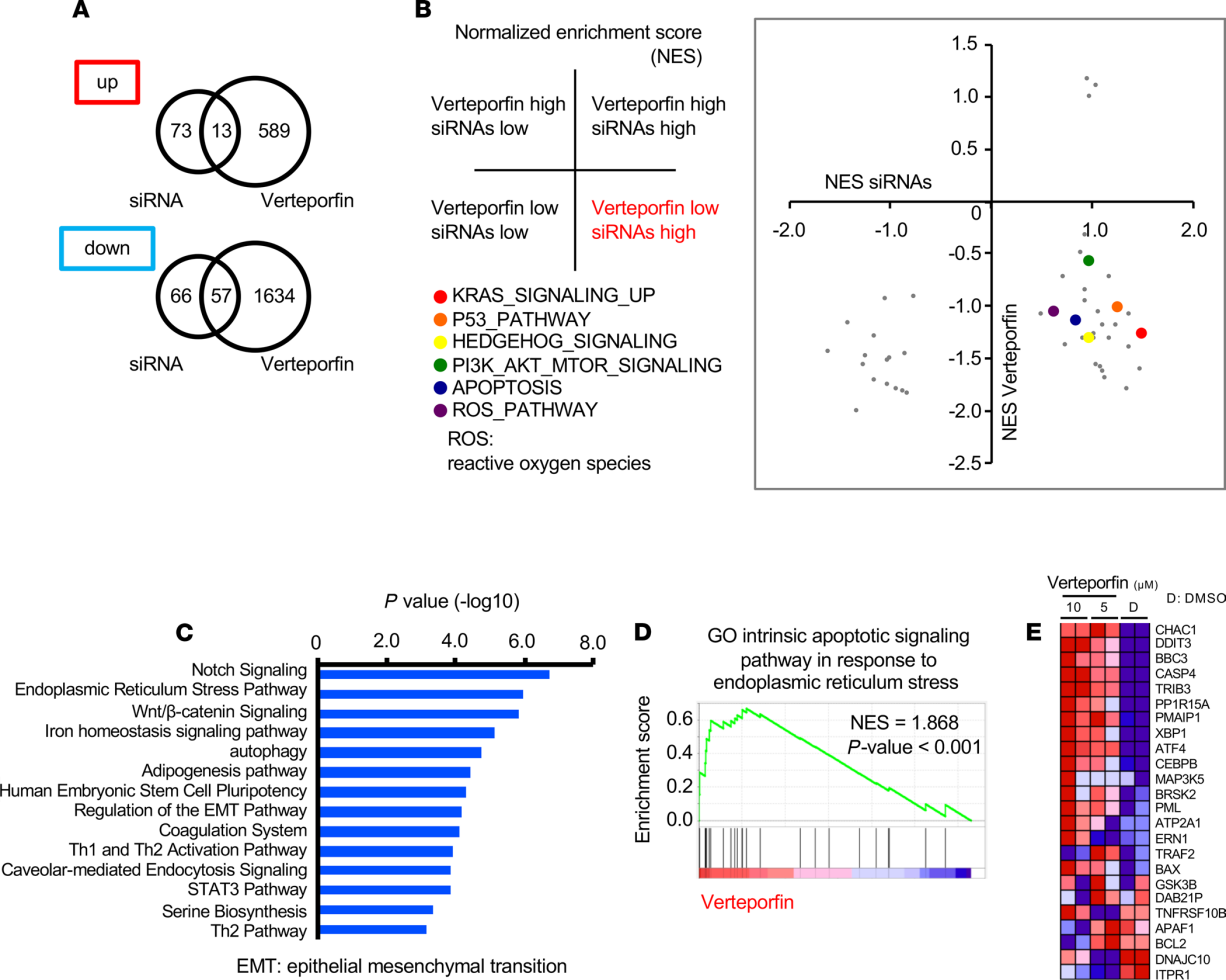
Verteporfin treatment is associated with the ER stress pathway. (**A**) Venn diagram showing upregulated genes and downregulated genes following treatment with YAP1 siRNAs and verteporfin at various concentrations. (**B**) Correlation of the normalized enrichment scores following verteporfin treatment and YAP1 knockdown from GSEA of all hallmark gene sets. (**C**) Related pathways and functional annotation of genes specifically altered by verteporfin treatment analyzed by ingenuity pathway analysis (QIAGEN). (**D**) GSEA to assess apoptotic signaling in response to ER stress in A-549 cells treated with various concentrations of verteporfin. GO, Gene Ontology. The *P* values in the graphs were calculated by GSEA. (**E**) Heatmap of leading-edge genes in the ER stress pathway.

Differentially expressed genes only partially overlapped, and most changes in gene expression were independently altered due to verteporfin treatment (Figure 5A). Most major cancer-related pathways, such as the TP53, KRAS, and apoptosis pathways, were downregulated by only verteporfin treatment (Figure 5B). We further examined the impact of verteporfin and YAP1 knockdown on RAS-related proteins. Most proteins were attenuated by verteporfin treatment but behaved differently by siRNA YAP1 knockdown Supplemental Figure 5, A and B). To elucidate the molecular mechanism underlying the observed antitumor effects of verteporfin, we examined the different gene expression patterns by IPA, which demonstrated that the most frequently involved canonical pathways were Notch signaling, the ER stress pathway, and Wnt/β-catenin signaling (Figure 5C). Also, GSEA of the verteporfin treatment revealed significant enrichment of apoptotic signaling pathway in response to ER stress (Figure 5D). Interestingly, the expression of known ER stress pathway genes, such as DNA damage-inducible transcript 3 (*DDIT3*), tribbles pseudokinase 3 (*TRIB3*), protein phosphatase 1 regulatory subunit 15A (*PPP1R15A*), and activating transcription factor 4 (*ATF4*), was altered in response to verteporfin treatment (Figure 5E). Together, these data suggest that verteporfin treatment induced ER stress in KRAS-mutant lung cancer cells.

### Verteporfin causes unresolved ER stress specific to KRAS-mutant lung cancer cells.

**Figure 6:**
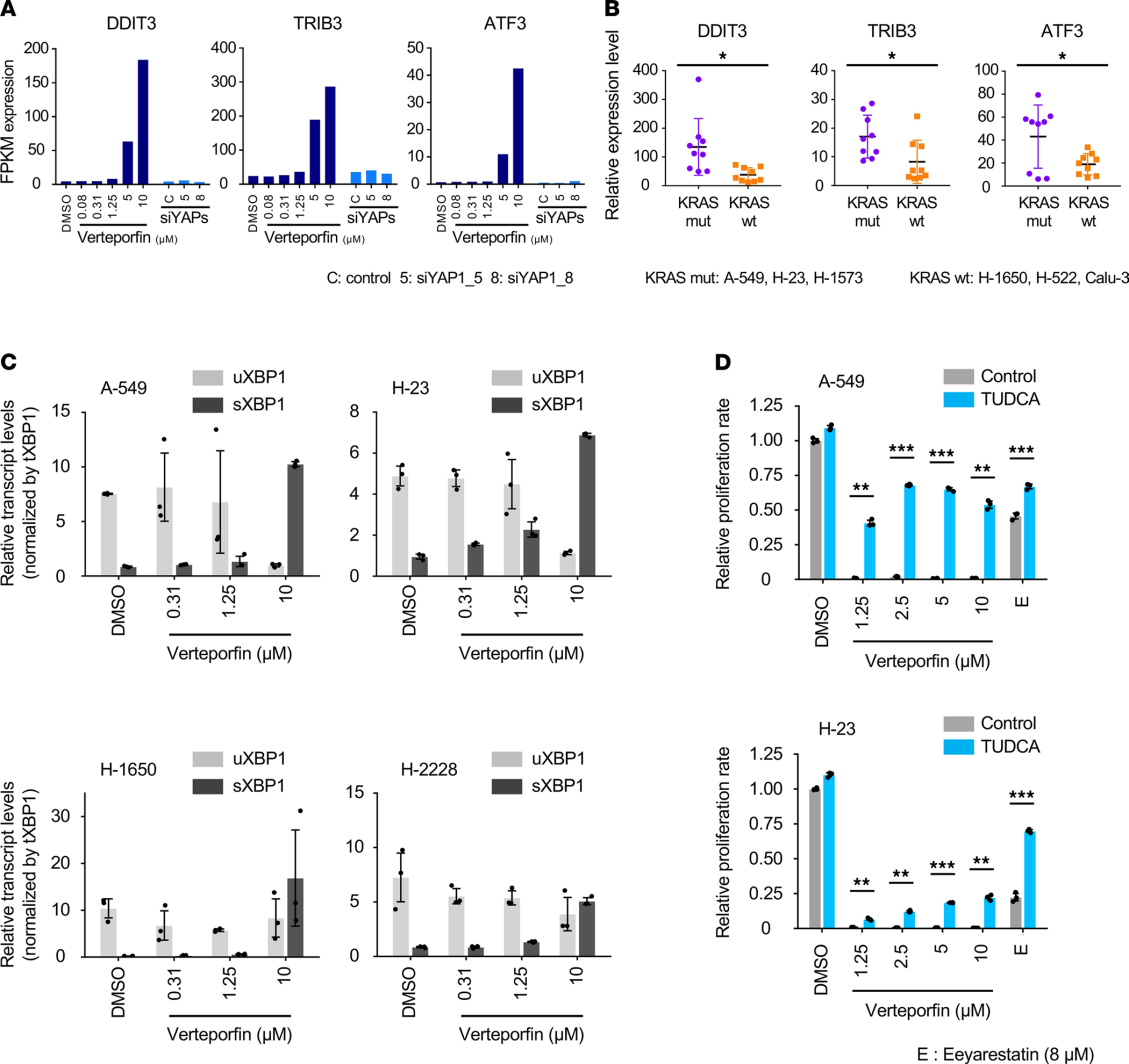
Characterization of unresolved ER stress mechanism in KRAS-mutant lung cancer cells by verteporfin treatment. (**A**) Relative expression analysis of leading genes in ER stress pathways in KRAS-mutant cells treated with verteporfin or YAP1 knockdown by RNA-Seq. (**B**) Relative expression analysis of leading genes in ER stress pathways in KRAS-mutant and WT cells treated with verteporfin (control and 10 μM) determined by quantitative reverse transcription PCR. The values are the mean ± SD (3 KRAS-mutant cell lines [A-549, H-23 and H-1573] and 3 WT cell lines [H-1650, H-522 and Calu-3], each *n* = 3). Statistical significance was determined using an unpaired 2-tailed Student’s *t* test. *, *P* < 0.05. (**C**) Characterization of unspliced XBP1 (uXBP1) and spliced XBP1 (sXBP1) in KRAS-mutant and WT cells treated by verteporfin in various concentrations. The normalization control was performed by total XBP1 (tXBP1). The values are the mean ± SD (*n* = 3). (**D**) Effects of combination therapy of verteporfin and TUDCA (ER stress inhibitor) in KRAS-mutant cells. The results of combination therapy of eeyarestatin (ERAD inhibitor) and TUDCA are shown as a control. E, eeyarestatin. The values are the mean ± SD (*n* = 3). Statistical significance was determined using an unpaired 2-tailed Student’s *t* test. **, *P* < 0.01 and ***, *P* < 0.001. FPKM, fragments per kilobase per million mapped reads.

To corroborate the presence of unresolved ER stress, we have analyzed the encoded genes that play a key role in the ER stress pathway. The results showed adverse alterations in expression by verteporfin treatment compared with the baseline condition, while YAP1 knockdown had almost no effect (Figure 6A and Supplemental Figure 6, A and B). We next explored the difference in the effects of verteporfin treatment between KRAS-mutant and WT cells. Quantitative reverse transcription PCR targeting the ER stress pathway genes showed the significant upregulation of *DDIT3*, *TRIB3*, and *ATF3* in KRAS-mutant cells compared with WT cells (Figure 6B and Supplemental Figure 6C). These data suggested that KRAS-mutant lung cancer cells are targeted by verteporfin via unresolved ER stress pathway, which could lead to ER stress–induced cell death. To further characterize the ER stress–induced mechanism following verteporfin treatment, we validated the downstream effector of the ER stress pathway. Inositol-requiring enzyme-1–mediated (IRE1-mediated) X-box binding protein 1 (XBP1) splicing, one of the main unfolded protein response (UPR) markers of the ER stress response, was promoted by verteporfin treatment in KRAS-mutant cells specifically (Figure 6C). Moreover, the eukaryotic translation initiation factor 2 subunit alpha (eIF2α) phosphorylation was induced by verteporfin treatment in a dose-dependent manner in KRAS-mutant cells (Supplemental Figure 6D). Together, these data indicate the involvement of UPR markers by verteporfin treatment in KRAS-mutant cells. Finally, we show that the mitigation of verteporfin-induced cell death in KRAS-mutant cells can be achieved by tauroursodeoxycholic acid (TUDCA, ER stress inhibitor) administration (Figure 6D). The activity of TUDCA as an ER stress inhibitor was confirmed by combination with eeyarestatin (ER-associated degradation [ERAD] inhibitor, triggers ER stress) (Figure 6D). These data convinced us that the predominant ER stress mechanism underlying cell survival in verteporfin-treated KRAS-mutant lung cancer was mainly explained by IRE1-mediated XBP1 splicing.

## Discussion

Despite its remarkable impact on the therapeutic approach in lung cancer, targeting KRAS-mutant lung cancer cells remains a challenge; however, KRAS-G12C–specific inhibitors at the clinical trial stage are forthcoming (26, 27). Here, we identified verteporfin, which is a known YAP1 inhibitor, as a striking therapeutic candidate for KRAS-mutant lung cancer cells involving the unresolved ER stress mechanisms. Many groups have described the potential antitumor effect of verteporfin, including that in KRAS-mutant cancer cells. However, the molecular mechanism underlying the effectiveness of verteporfin in KRAS-mutant lung cancer cells remains obscure. Our comparative approach using small-molecule compounds and gene silencing enabled an improved understanding of cytotoxic status during apoptosis.

Systematic screening with a chemical-based approach has produced a therapeutic candidate in KRAS-mutant lung cancer cells. Verteporfin, a known YAP1 inhibitor, has received a great deal of interest as a potential therapeutic modality for various cancers. Consistent with previous reports (28, 29), our data showed its inhibitory effects on cell proliferation in KRAS-mutant lung cancer cells. The essential role of the Hippo pathway and YAP1 in various types of epithelial tumors is well established, and YAP is amplified in many human cancers. Moreover, recent studies reported the antineoplastic activity of verteporfin in various carcinomas, such as myxoid liposarcomas, pancreatic carcinomas, bladder cancers, and synovial sarcomas (16, 30–32). The Hippo pathway and especially YAP activity are linked with oncogenic *RAS*. YAP was reported to function as a critical partner of mutant *KRAS* in pancreatic ductal adenocarcinoma (PDAC) (33), and the potential of the YAP1 oncogene as a KRAS-independent bypass mechanism has been described in PDAC tumor cells (34). Specific EMT regulation induced by YAP1 with oncogenic KRAS was shown in a murine lung cancer model (35). Therefore, we assumed that the inhibitory effects of verteporfin in KRAS-mutant lung cancer cell proliferation were related to EMT-related mechanisms, but we did not observe a clear relationship between the effects of verteporfin and EMT in this study. This raises the question of whether verteporfin truly functions as an inhibitor of YAP1. Given the complexity of biochemical pathways and the number of protein interactions now known, the treatment effect of verteporfin was not confirmed to be the direct inhibition of YAP itself, consistent with previous reports (17, 36). To this end, we performed comparable experiments under conditions of verteporfin treatment and YAP1 siRNA knockdown.

We examined the data following 2 independent treatments. First, KRAS-mutant lung cancer cells underwent verteporfin treatment; KRAS-mutant lung cancer cells also underwent siRNA-mediated knockdown of YAP1. Cell proliferation assays showed the significant inhibitory effects of verteporfin treatment but not YAP1 knockdown. Hippo pathway-related protein expression levels were differentially affected by the 2 treatments, as shown by Western blot analysis. Based on these data, we next carried out RNA-Seq analysis using RNA extracted from cells treated with verteporfin and YAP-knockdown cells. The RNA-Seq data revealed various cancer-related signaling pathways specifically involved in verteporfin treatment, such as Notch signaling, Wnt/β-catenin signaling, and the ER stress pathway, which is consistent with the previous reports (37, 38). Because several genes, including *DDIT3*, *TRIB3*, and *ATF3*, were specifically increased due to verteporfin treatment in KRAS-mutant lung cancer cells, we focused on the ER stress pathway. Previous work has implicated the oncogene *KRAS* as a regulator of cancer metabolism that orchestrates several metabolic pathways, including ER stress pathway (39, 40). The ER stress pathway, a critical signaling and metabolic pathway for cancer homeostasis characterized by the accumulation of unfolded proteins and the UPR, is related to cellular homeostasis and cell death (41, 42). Understanding of the mechanisms that regulate cancer cells under tumorigenic conditions may provide insights into their malignant potency, and this pathway has been well documented in most major types of human cancer (43). However, whether this stress response is associated with only KRAS-driven lung tumorigenesis is unknown. ER stress is controlled by 3 major ER-resident membrane proteins, IRE1α (encoded by *ERN1*), ATF6 (encoded by *ATF6*), and protein kinase RNA-like ER kinase (PERK) (encoded by *EIF2AK3*) (44–46). When stress occurs, protein complexes between the ER-resident chaperone binding immunoglobulin protein and these 3 transmembrane signaling molecules will interact (47). Our present work shows that the specific IRE1-mediated XBP1 splicing was induced drastically by verteporfin treatment in KRAS-mutant cells. In addition to discovering the involvement of the major sensor mechanism in the ER stress pathway, eIF2α phosphorylation, one of the classical ER stress–related responses acting downstream of PERK-eIF2α-ATF4-C/EBP homologous protein signaling, was induced by verteporfin treatment in a dose-dependent manner. These observations suggest the activation of the UPR by verteporfin treatment, and this effect was attenuated by TUDCA, a chemical chaperone broadly used as an ER stress inhibitor. Taken together, our results highlighted the potentially novel mechanism of verteporfin, a critical regulator, in KRAS-mutant lung cancer cells, which involves unresolved ER stress.

In conclusion, our data showed the clear effects of verteporfin as a therapeutic modality in KRAS-mutant lung cancer cells. Although verteporfin is a small molecule demonstrated to inhibit TEAD-YAP interactions and suppressed YAP-induced carcinogenesis, many other gene regulatory mechanisms, including unresolved ER stress, are also involved. The continued study of targeting a specific vulnerability of KRAS-mutant lung cancer cells may provide new insights for therapeutic intervention that warrant further investigation.

## Methods

### Cell culture.

Human lung cancer cell lines (A-549, H-23, H1573, H-1373, H-1734, H-2347, H-2444, H-1650, H-522, Calu-3, H-1395, H-1435, H-1838, H-2228, H-2286, A-427) were purchased from American Type Culture Collection (ATCC). A-549 and Calu-3 cells were maintained and passaged in Dulbecco’s modified Eagle medium (DMEM) (Gibco, Thermo Fisher Scientific) supplemented with 10% fetal bovine serum (FBS) (Gibco, Thermo Fisher Scientific) and 1% antibiotic-antimicotic solution (Gibco, Thermo Fisher Scientific) in a humidified incubator at 37°C and 5% CO_2_. H-23, H1573, H-1373, H-1734, H-2347, H-2444, H-1650, H-522, H-1395, H-1838, H-2228, and A-427 cells were maintained and passaged in Roswell Park Memorial Institute–1640 medium (Gibco, Thermo Fisher Scientific) supplemented with 10% FBS (Gibco, Thermo Fisher Scientific) and 1% antibiotic-antimicotic solution (Gibco, Thermo Fisher Scientific) in a humidified incubator at 37°C and 5% CO_2_. H-1435 and H-2286 cells were maintained and passaged in DMEM/nutrient mixture F12 (Gibco, Thermo Fisher Scientific) supplemented with 10% FBS and 1% antibiotic-antimicotic solution in a humidified incubator at 37°C and 5% CO_2_. The summary of signature genetic changes of the cell lines is listed in Supplemental Table 3.

### Drug treatment.

The Prestwick Chemical Library was purchased from Prestwick Chemical. This library contains 1271 small molecules consisting of 95% approved drugs (by the FDA, European Medicines Agency, and other agencies). Verteporfin was obtained from Cayman Chemical Company. TUDCA was obtained from Selleck. Eeyarestatin 1 was obtained from Cayman Chemical Company. Drugs were dissolved in DMSO for each analysis.

### Cell proliferation assay.

Cell proliferation was evaluated with CellTiter-Glo 2.0 reagent (Promega) as described by the manufacturer’s instructions. Cells were seeded onto a 96-well white plate at 5.0 × 10^3^ cells/well. Six hours after seeding, drugs were added at a concentration of 10 μM. After 48 hours in the case of A-549 cells and after 72 hours in the case of other cell lines, cell viability was measured with CellTiter-Glo 2.0 reagent. Luminescence measurements were taken with a microplate reader 10 minutes after the agent was added (BioTek, Gen5 Synergy H4).

### Apoptotic assay.

To evaluate apoptosis, a luminescent caspase-3/7 activation assay was performed. The cells were seeded in a white 96-well plate, and after 6 hours of incubation, 10 μM of the selected drugs was added. After incubation for 48 to 72 hours, Caspase-Glo reagent (Caspase-Glo 3/7 assay; Promega) was added and incubated for 1 hour, and the activity of caspase-3/7 was measured using a microplate reader (BioTek).

### Western blot analysis.

The cells were gently scraped from the culture plates, resuspended in 1000 μL of Mammalian Protein Extraction Reagent buffer (Thermo Fisher Scientific), and shaken for 5 minutes. The samples were then centrifuged at 14,000*g* for 10 minutes. The supernatants were collected, and the protein concentration was calculated using a Qubit 2.0 fluorometer (Thermo Fisher Scientific). Protein extracts (30 μg per lane) were prepared, run on a 4%–20% Mini-PROTEAN TGX gel (Bio-Rad), and transferred to a 0.45 μm PVDF membrane. The membranes were blocked for 1 hour at room temperature using Blocking One (nacalai tesque), followed by incubation overnight at 4°C with the primary antibodies presented in Supplemental Table 1. Two secondary antibodies — anti-mouse IgG, HRP-linked whole Ab sheep (GE Healthcare, now Cytiva, NA931-1ML), and anti-rabbit IgG, HRP-linked antibody (Cell Signaling Technology, 7074S) — were used at a dilution of 1:5000, and the membranes were developed using ImmunoStar LD (Wako) and imaged using the FUSION Solo 7S (Vilber-Lourmat).

### Immunohistochemistry.

Harvested tumors were fixed in 4% paraformaldehyde overnight and embedded in paraffin. Tumor sections were dewaxed with xylene and rehydrated with ethanol (100%–70%). Antigen retrieval was performed by boiling the specimens in Immunosaver (Nissin EM) diluted 1:200 for 45 minutes at 98°C. Endogenous peroxidase activity was quenched by incubation with 3% H_2_O_2_ for 30 minutes, and the sections were permeabilized with 0.1% Triton X-100 (MilliporeSigma) for 15 minutes. After blocking with Dako blocking reagent for 30 minutes, sections were incubated with primary antibodies overnight at 4°C in a humidified box. Sections were then incubated with secondary antibodies with ImPRESS IgG-peroxidase kits (Vector Labs) and DAB chromogen and counterstained with hematoxylin. Stained sections were imaged using a BZ-X700 microscope (Keyence).

### Quantitative reverse transcription PCR.

Total RNA was collected from cancer cells using the RNeasy Mini Kit (250, QIAGEN) according to the manufacturer’s instruction. cDNA was synthesized with a High-Capacity cDNA Reverse Transcription Kit (Thermo Fisher Scientific) using an Applied Biosystems StepOnePlus system (Thermo Fisher Scientific). Sequences of the PCR primers used are listed in Supplemental Table 2.

### RNA-Seq.

Total RNA was extracted from A-549 cells treated with verteporfin and YAP1-knockdown A-549 cells using RNeasy Mini Kit (250, QIAGEN) according to the manufacturer’s protocol. After amplification of the total RNA, RNA quantity and quality were evaluated with a NanoDrop ND-1000 spectrophotometer (Thermo Fisher Scientific) and an Agilent Bioanalyzer (Agilent Technologies). Paired-end sequencing with a read length of 50 bases was performed on the Illumina NovaSeq 6000 platform following the manufacturer’s instructions. Raw RNA-Seq data were subjected to FastQC quality control. The sequencing data were analyzed by using the software packages of TopHat and mapped against hg19. Expression quantified by normalization of the number of reads was defined as FPKM by Cufflinks.

### Accession numbers.

Data generated from this study have been deposited in the DDBJ Sequence Read Archive (DRA) under accession number DRA009992.

### Animal studies.

Five-week-old female BALB/C nude mice were obtained from Charles River Laboratories. A-549 cells (KRAS-mutant) and H-1650 cells (WT) were injected into the right flanks of the mice with Matrigel/PBS (1.0 × 10^6^ cells, 50% final concentration) to establish xenograft models. Five days after inoculation, mice were randomly divided into 2 groups (*n* = 6/group) and treated with vehicle alone (olive oil with 3% DMSO) or verteporfin (50 mg/kg), which was injected intraperitoneally twice a week. Mice were monitored carefully and the size of their tumors was measured using a Vernier caliper. Tumors were harvested 25 days after the inoculation of cancer cells and tumor weight was measured. Blood samples were corrected for biochemical tests.

### Statistics.

The data are presented as the mean ± SD. Statistical significance between the 2 groups was determined using 2-tailed Student’s *t* test and among more than 3 groups using Dunnett’s multiple-comparison test. Differences with a *P* < 0.05 were considered significant.

### Study approval.

Animal studies were approved by the National Cancer Center Research Institute, Institute of Laboratory Animal Research (number: T18-009-M01).

## Author contributions

IS, NW, TY, MK, and YY designed, performed, and analyzed data from experiments. YT and KT designed experiments and helped with critical advice and discussion. IS and YY wrote the manuscript. The manuscript was finalized by TO with the assistance of all the authors. All authors read and approved the final manuscript.

## Supplementary Material

Supplemental data

## Figures and Tables

**Table 1:**
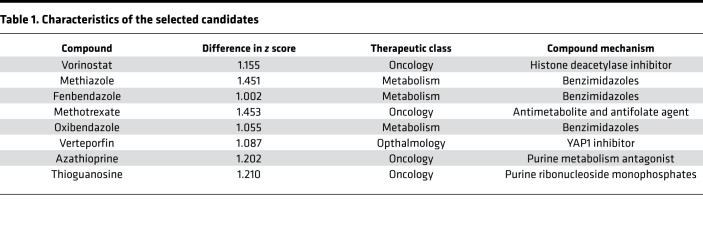
Characteristics of the selected candidates

## Version 1 04/08/2021

Electronic publication
