# Supplementary material for: Selective targeting of KRAS-driven lung tumorigenesis via unresolved ER stress
Source: JCI Insight. 2021 Apr 8;6(7):e137876. doi: 10.1172/jci.insight.137876 (PMC8119185; doi:10.1172/jci.insight.137876)
Supplement: Supplemental data [file jciinsight-6-137876-s067.pdf]

Supplemental data

Supplementary Table 1 Shimomura I et al.,

Antibody	Catalog number	Dilution	Company
Phospho-B-Raf (Ser445) Antibody	2696	1:1,000	CST, USA
B-Raf (55C6) Rabbit mAb	9433	1:1,000	CST, USA
Phospho-C-Raf (Ser338) (56A6) Rabbit mAb	9427	1:1,000	CST, USA
C-Raf (D5X6R) Mouse mAb	12552	1:1,000	CST, USA
Phospho-EGF Receptor (Tyr1068) (D7A5) XP Rabbit mAb	3777	1:1,000	CST, USA
EGF Receptor (D38B1) XP Rabbit mAb	4267	1:1,000	CST, USA
Phospho-Akt (Ser473) (D9E) XP Rabbit mAb,	4060	1:2,000	CST, USA
Akt (pan) (C67E7) Rabbit mAb	4691	1:1,000	CST, USA
Phospho-p44/42 MAPK (Erk1/2) (Thr202/Tyr204) (D13.14.4E) XP Rabbit mAb	4370	1:2,000	CST, USA
p44/42 MAPK (Erk1/2) (137F5) Rabbit mAb	4695	1:1,000	CST, USA
Phospho-MEK1/2 (Ser217/221) (41G9) Rabbit mAb	9154	1:1,000	CST, USA
MEK1/2 Antibody	9122	1:1,000	CST, USA
Phospho-p38 MAPK (Thr180/Tyr182) (D3F9) XP Rabbit mAb	4511	1:1,000	CST, USA
p38 MAPK (D13E1) XP Rabbit mAb	8690	1:1,000	CST, USA
Phospho-SAPK/JNK (Thr183/Tyr185) 81E11 Rabbit mAb	4668	1:1,000	CST, USA
SAPK/JNK Antibody	9252	1:1,000	CST, USA
Phospho-NF-κB p65 (Ser536) (93H1) Rabbit mAb	3033	1:1,000	CST, USA
NF-κB p65 (D14E12) XP Rabbit mAb	8242	1:1,000	CST, USA
Stat1 Antibody	9172	1:1,000	CST, USA
Anti-Actin Antibody, clone C4	MAB1501R	1:5,000	Merck, Germany
Ajuba (D4D8P) Rabbit mAb	34648	1:1,000	CST, USA
Merlin (D3S3W) Rabbit mAb	12888	1:1,000	CST, USA
TAZ (D3I6D) XP Rabbit mAb	70148	1:1,000	CST, USA
YAP (D8H1X) XP Rabbit mAb	14074	1:1,000	CST, USA
Phospho-eIF2α (Ser51) (D9G8) XP Rabbit mAb	3398	1:1,000	CST, USA
eIF2α Antibody	9722	1:1,000	CST, USA

CST: Cell Signaling Technology

Supplemental Table 1. Primary antibodies for western blots.

Supplementary Table 2 Shimomura I et al.,

Gene name	Nucleotide sequence (5' - 3')
GAPDH FW	GAAGGTGAAGGTCGGAGT
GAPDH RV	GAAGATGGTGATGGGATTC
ASNS FW	CGACCAAAAGAAGCCTTCAG
ASNS RV	GCCATCATTGCATCATCAAC
ATF3 FW	TCGGAGAAGCTGGAAAGTGT
ATF3 RV	TCTGGAGTCCTCCCATTCTG
ATF4 FW	CCAACAACAGCAAGGAGGAT
ATF4 RV	GTGTCATCCAACGTGGTCAG
DDIT3 FW	GCGCATGAAGGAGAAAGAAC
DDIT3 RV	TCACCATTCGGTCAATCAGA
TRIB3 FW	TGGTACCCAGCTCCTCTACG
TRIB3 RV	GACAAAGCGACACAGCTTGA
PPP1R15A FW	GAGGAGGCTGAAGACAGTGG
PPP1R15A RV	AATTGACTTCCCTGCCCTCT
ATF6 FW	GCCTTTATTGCTTCCAGCAG
ATF6 RV	TGAGACAGCAAAACCGTCTG
ERN1 FW	CGTCCTGTGGATCCAAAACT
ERN1 RV	GTCAGATAGCGCAGGGTCTC
EIF2AK3 FW	GGCCACTTTGAACTTCGGTA
EIF2AK3 RV	GCAGCTTCCTGTTCTTCCAC
uXBP1 FW	CAGACTACGTGCACCTCTGC
uXBP1 RV	CTGGGTCCAAGTTGTCCAGAAT
sXBP1 FW	GCTGAGTCCGCAGCAGGT
sXBP1 RV	CTGGGTCCAAGTTGTCCAGAAT
tXBP1 FW	TGAAAAACAGAGTAGCAGCTCAGA
tXBP1 RV	CCCAAGCGCTGTCTTAACTC

FW: forward
RV: reverse

Supplemental Table 2. Primer sequences for qRT-PCR.

Supplementary Table 3 Shimomura I et al.,

gene / cell line	KRAS	RET	mTOR	EGFR	BRAF	TP53	SMARCA4	STK11	KEAP1	PTEN	FAT4
A-427	p.G12D				p.P341P						
H-1373	p.G12C	p.P560P				p.E339*					p.R1411S
H-1734	p.G13C					p.R273L		p.M51fs			
H-2444	p.G12V					p.Y236C				p.P96S	
H-2347	p.L19F	p.GP798fs				p.T125T					p.D4016E
A-549	p.G12S						p.QSYYAV AH729fs	p.Q37*	p.G333C		p.Y444*
H-23	p.G12C	p.R77L				p.M246I	p.K1566N, p.E1567*	p.W332*	p.Q193H		p.D1853Y, p.T4458T
H-1573	p.G12A	p.T492T				p.R248L	p.E1399*	p.S216F	p.A143P		p.A4507S
H-1395					p.G469A			p.L55fs			p.L2310I
H-1435						p.C141W	p.G1162C	p.S299F, p.S299S, p.I300I	p.R413L		p.E2630A, p.T3163T
H-1838						p.R273L	p.V1529V				
H-2228		p.A498V				p.Q331*					
H-2286						p.K291*	p.V1296V, p.R1135Q	p.D194H		p.G230V	p.R753S, p.I1789I
H-1650				p.ELREA70 1del			P.N1164Y				
H-522		p.A281V	p.T1837N, p.V1801G			p.P191fs	p.P270fs				
Calu-3						p.M237I					

p : protein change

Supplemental Table 3. The summary of signature genetic changes.

Supplementary Figure 1 Shimomura I et al.,

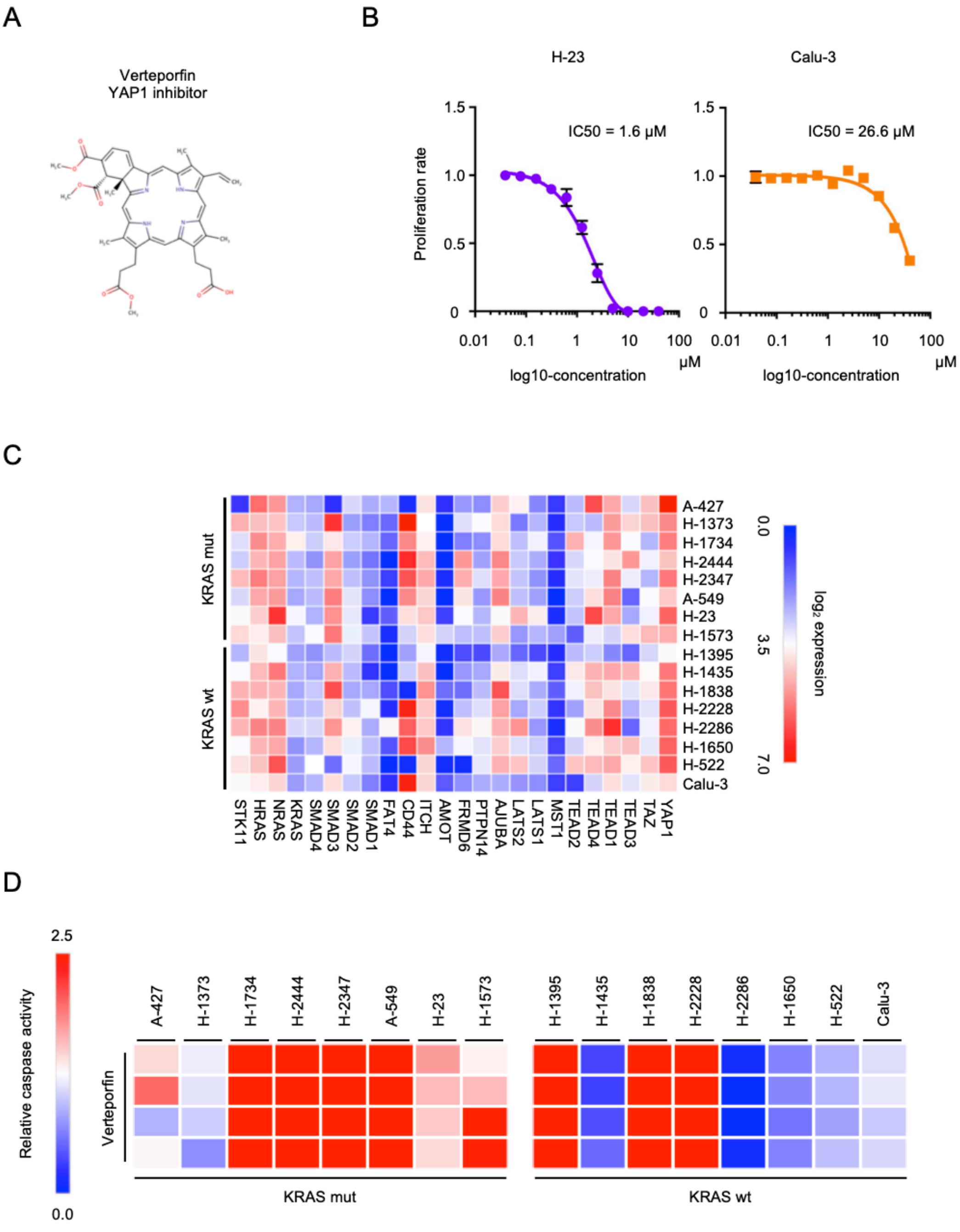

Supplemental Figure 1. Background of verteporfin and regarding the status of the cell lines.

A. The structure of verteporfin. **B.** Cell viability analysis in H-23 (KRAS-mutant) and Calu-3 (wild-type) cells after verteporfin treatment. **C.** The expression status of Hippo pathway-related genes in the cell lines. **D.** Relative caspase activity in all cell lines after verteporfin treatment.

Supplementary Figure 2 Shimomura I et al.,

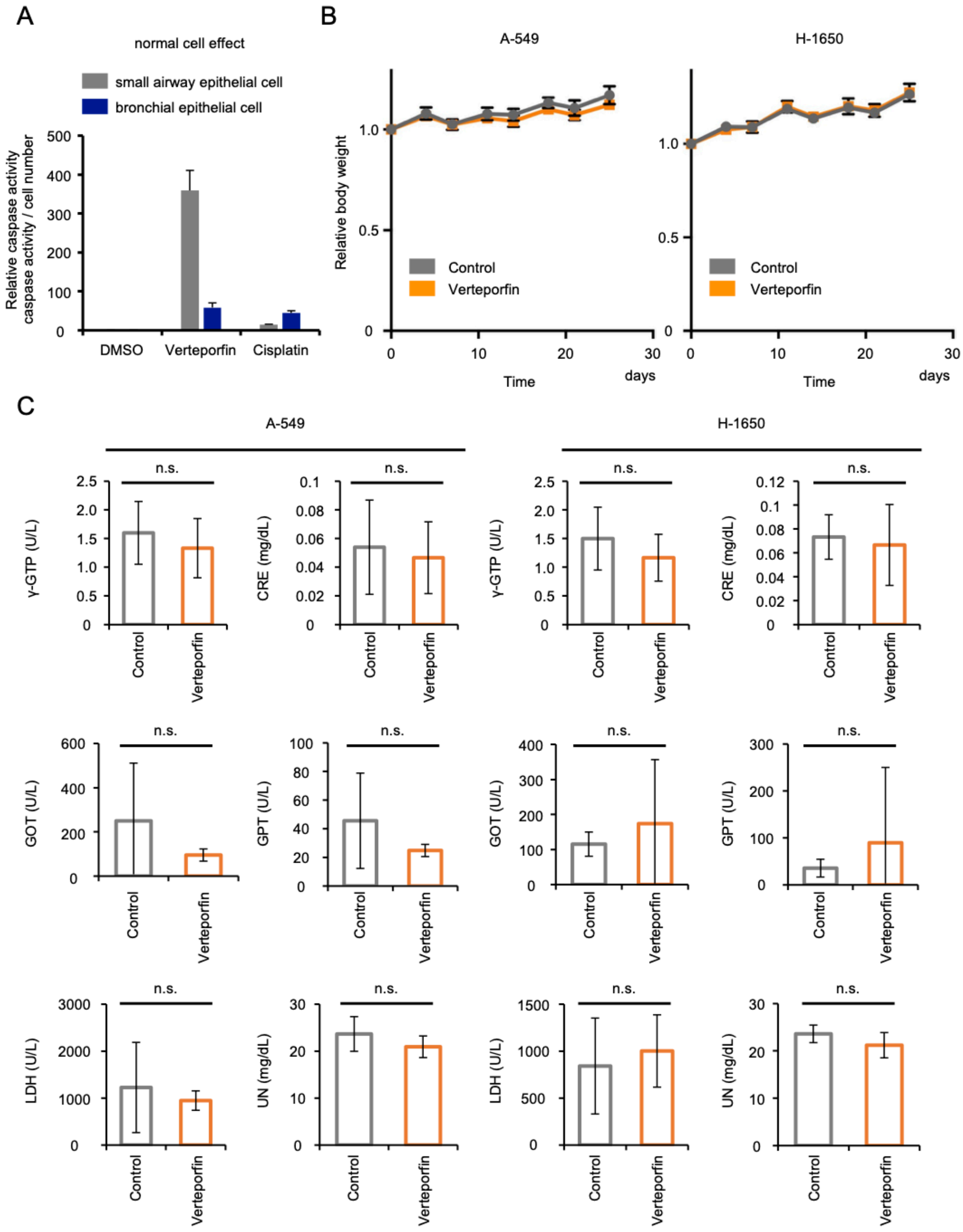

Supplemental Figure 2. Effects of verteporfin on normal cells and control mice.

A. Relative caspase activity in normal epithelial cells after treatment with DMSO, verteporfin and cisplatin. **B.** The body weights of the control and verteporfin treatment

groups of KRAS-mutant and wild-type cell xenograft mice. The values are the mean ± SD (n=6). C. Blood chemistry was analyzed at the time of euthanasia. The values are the mean ± SD (n=5 for the KRAS-mutant group and n=6 for the wild-type group). Statistical significance was determined using an unpaired 2-tailed Student's *t*-test. n.s., not significant.

Supplementary Figure 3 Shimomura I et al.,

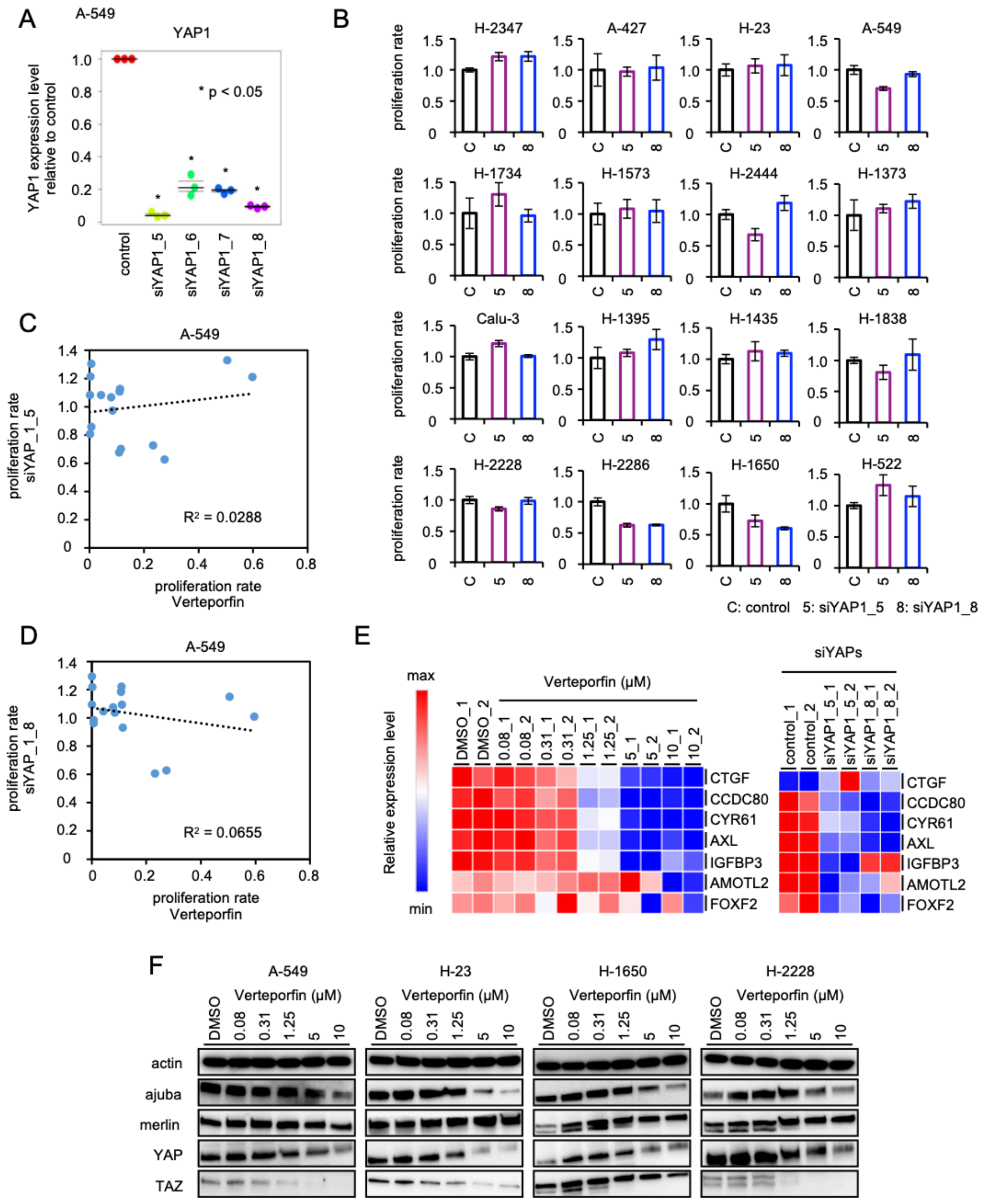

Supplemental Figure 3. Effect of YAP1 knockdown on cell lines.

A. The efficiency of YAP1 knockdown in A-549 cells as demonstrated by qRT-PCR. The values are the mean ± SD (n=3). Statistical significance was determined using Dunnett's

multiple comparison test. *, $p < 0.05$. **B.** Effect of YAP1 siRNAs on the proliferation of all cell lines. The values are the mean ± SD (n=3). **C, D.** Correlation of changes in cell proliferation following YAP1 knockdown and verteporfin treatment. **E.** The expression of the major YAP/TAZ transcriptional genes in A-549 cells (KRAS-mutant) treated by verteporfin and YAP siRNAs based on the whole-transcriptomic analysis. **F.** Western blotting for evaluating the expression levels of the Hippo pathway-related proteins in KRAS-mutant and wild-type cells by verteporfin treatment in various concentration.

Supplementary Figure 4 Shimomura I et al.,

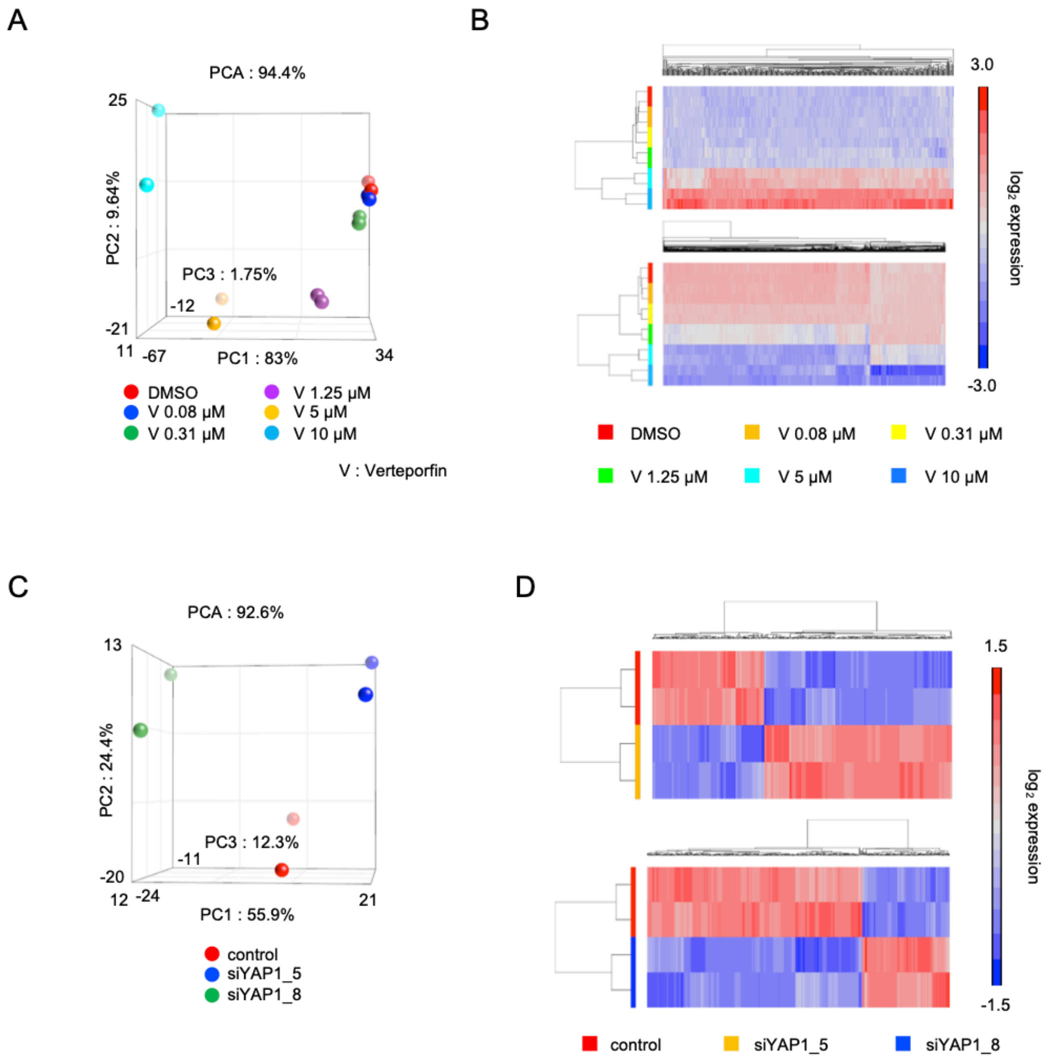

Supplemental Figure 4. Gene expression profile of verteporfin treatment and YAP1 knockdown.

A. PCA of A-549 cells treated with verteporfin at various concentrations. **B.** Heatmap showing genes that were downregulated and upregulated following treatment with verteporfin at various concentrations. **C.** PCA of A-549 cells treated with YAP1 siRNAs. **D.** Heatmap showing genes that were downregulated and upregulated by YAP1 knockdown.

Supplementary Figure 5 Shimomura I et al.,

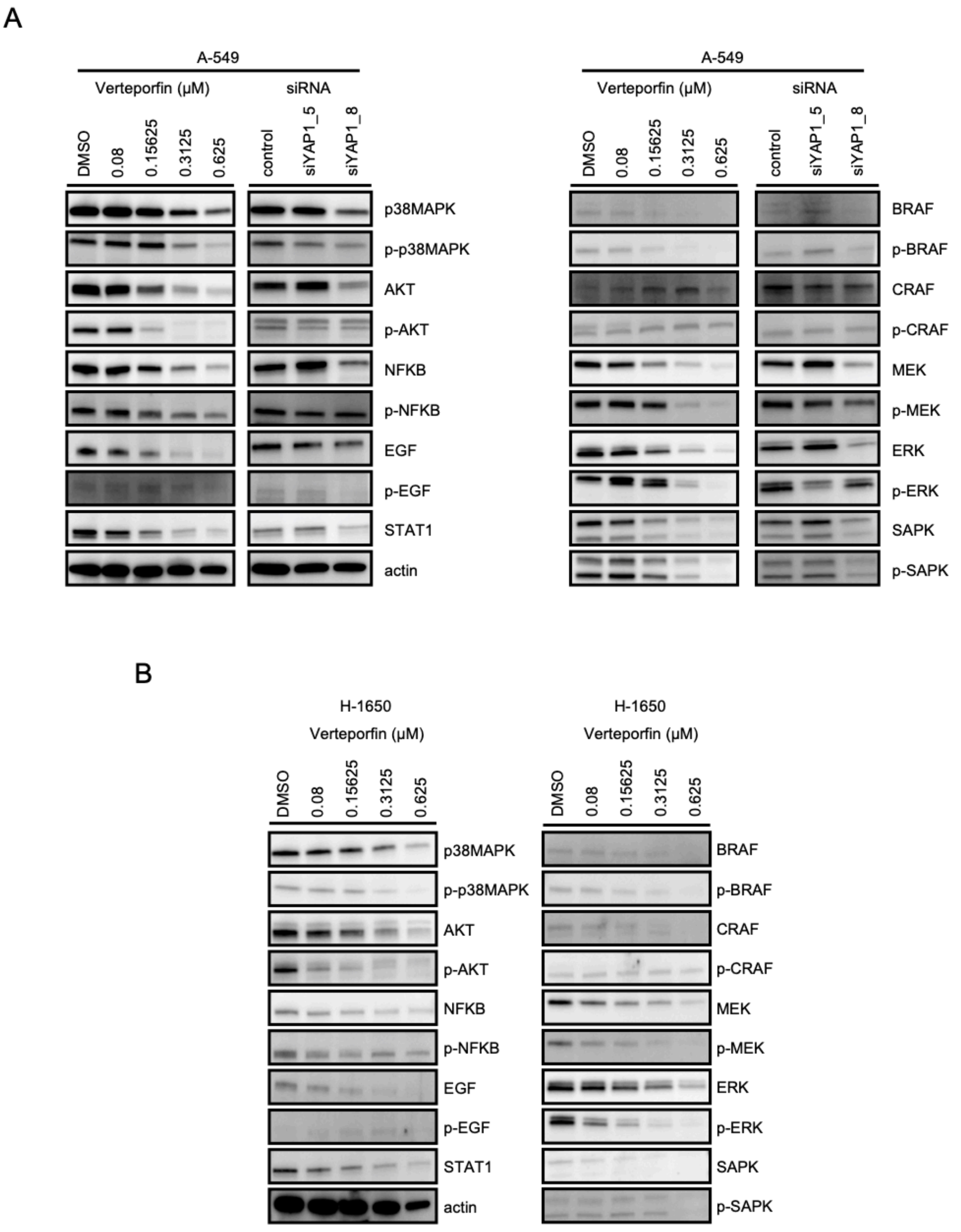

Supplemental Figure 5. Effect of verteporfin and YAP1 knockdown in RAS-related protein expressions.

A. Western blotting for changes in proteins in RAS-related pathways following treatment with increasing doses of verteporfin and YAP1 knockdown in KRAS-mutant cells. **B.** Western blotting for dose-dependent changes in the expression of proteins in RAS-related pathways in KRAS wild-type cells following treatment with verteporfin.

Supplementary Figure 6 Shimomura I et al.,

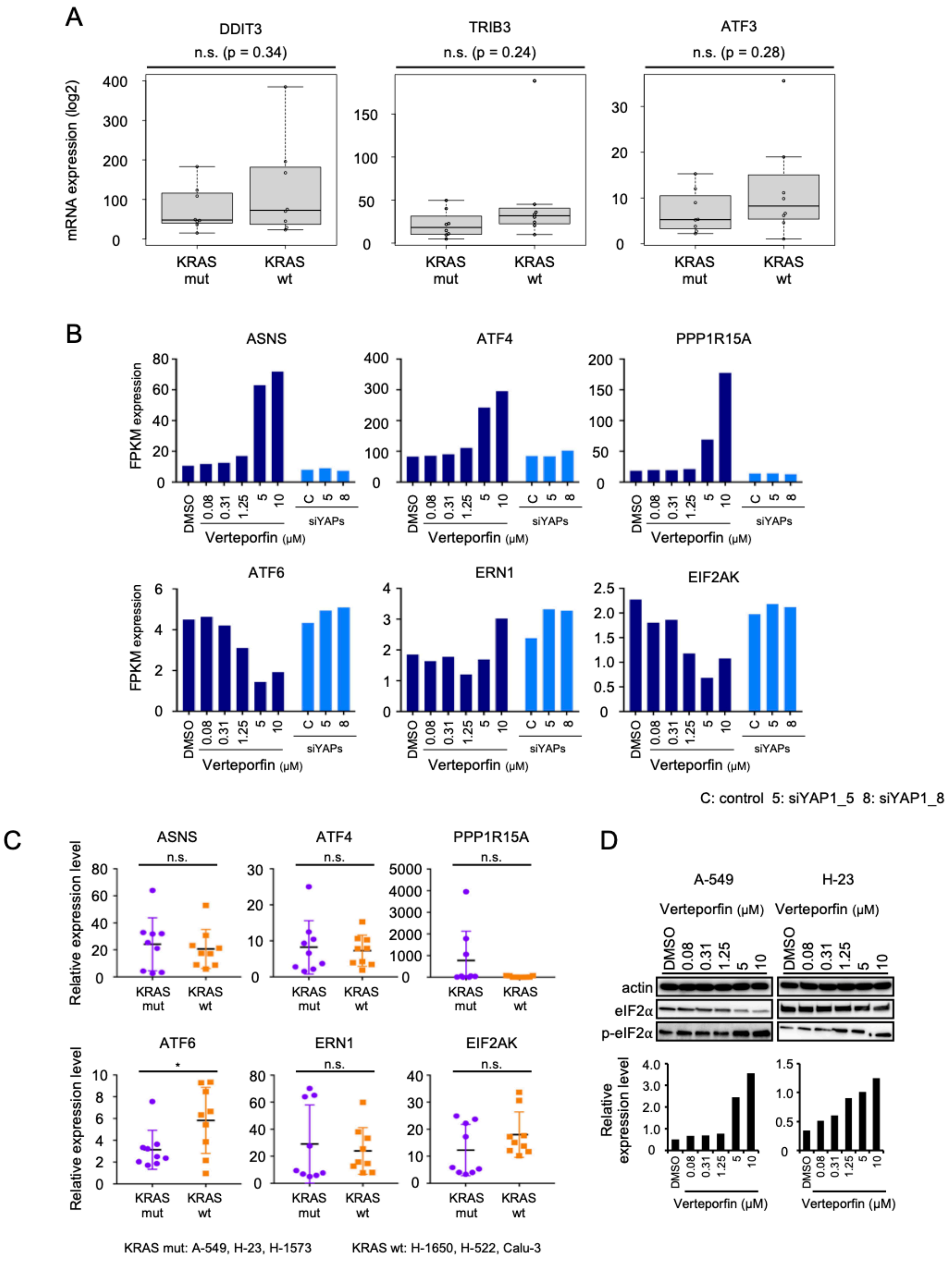

Supplemental Figure 6. Effect of verteporfin on replication of genes related to ER stress in KRAS-mutant and wild-type cells.

A. The expression of DDIT3, TRIB3 and ATF3 in KRAS-mutant (8 cell lines) and wild-type (8 cell lines) cells were analyzed from the data of CCLE. Statistical significance was determined using an unpaired 2-tailed Student's *t*-test. n.s., not significant. **B.** Relative expression analysis of genes involved in ER stress pathways in KRAS-mutant cells treated with verteporfin or YAP1 knockdown determined by RNA-seq. **C.** Relative expression analysis of genes involved in ER stress pathways in KRAS-mutant and wild-type cells treated with verteporfin (control and 10 μM) determined by qRT-PCR. The values are the mean ± SD (3 KRAS-mutant cell lines (A-549, H-23 and H-1573) and 3 wild-type cell lines (H-1650, H-522 and Calu-3), each n=3). Statistical significance was determined using an unpaired 2-tailed Student's *t*-test. *, $p < 0.05$ and n.s., not significant. **D.** Western blotting for evaluating the protein expression levels of the eIF2α and phosphorylated eIF2α in KRAS-mutant cells by verteporfin treatment in various concentration.
